# Making the transition: A perspective from two PhDs in the US

**DOI:** 10.1007/s40037-020-00582-4

**Published:** 2020-05-07

**Authors:** Tasha R. Wyatt, Kelsey Larsen

**Affiliations:** 1 Medical College of Georgia, Augusta University, Augusta, USA; 2 Uniformed Services University of the Health Sciences, Bethesda, MD USA

The work of Etmansky and colleagues highlights the interdisciplinary world of medical education, with particular emphasis on the negotiation that occurs among disciplines grounded in different methodologies, assumptions, and orientations [1]. At this intersection, the ‘traditional metrics’ of success no longer apply and require individuals think differently about their activities.

As medical education researchers from two distinctly different fields, we recognize the thematic undercurrent of *negotiation *and its trans-national commonality. We have also experienced the assumptions, conventions, and adaptations needed to work in this interdisciplinary field, and experienced the tension of what it means to be successful in our home disciplines and medical education. Thus, in the same spirit of narrative sensemaking that grounds Etmansky et al.’s research, we wanted to share our narratives of negotiation to highlight how we navigate these tensions.

## Tasha’s narrative

My transition into medical education was an existential and epistemological “crisis.” As an ally of indigenous communities, I had not worked in a field that embraced positivist research, and my previous training didn’t prepare me for the depths of professional discomfort I experienced as a critical constructivist. Initially, I struggled to understand how I could embrace positivism, but eventually realized that PhD researchers outside the US included their epistemologies and theoretical lenses in their work; their voices were clear, resonant, and not just limited to a reflexive statement or choice of analytical lens. By contrast, PhDs in the US are often expected to perform discrete tasks (e.g. develop assessments, conduct evaluations, support scholarship). These tasks are essential, but garner feelings of liminality as we wonder about a future point where our disciplinary training might matter.

## Kelsey’s narrative

As a political scientist, I had two key assumptions: medical education understood the dedicated methodological pluralism that defines a modern political scientist’s skills, and that all disciplines defined faculty success the same way—as a product of exemplary research, teaching, and service. I joined medical education under the presumption that we were all a product of *the* academy, not *individual* academies. Medical education challenged those assumptions, and forced me to develop negotiation skills to support my research. I have learned to seek out internal and external collaborators who are open to my discipline-specific abilities and interested in incorporating them. I have also adjusted my definition of faculty success by relying on colleagues and mentors to provide guidance and adaptation.

Etmansky et al.’s work highlights this negotiation process among PhDs working in Canadian research units. However, even as US-based PhDs, their work deeply resonated with us and our experiences as outside researchers joining medical education.
